# A case report: Diagnosis and treatment of idiopathic hypertrophic pachymeningitis

**DOI:** 10.1002/ibra.12099

**Published:** 2023-04-02

**Authors:** Zhong Luo, Piao Cao, Jing‐Qing Xu, Rong Yan, Jian Wang, Tao Liang, Ya Chen, Zu‐Cai Xu

**Affiliations:** ^1^ Department of Neurology Affiliated Hospital of Zunyi Medical University Zunyi China; ^2^ Department of Neurology, Guizhou Aerospace Hospital Zunyi China

**Keywords:** Autoimmune disease, Headache, Idiopathic hypertrophic pachymeningitis (IHP), Magnetic resonance imaging (MRI), Myelin oligodendrocyte glycoprotein (MOG)

## Abstract

A 57‐year‐old man who suffered from a headache for 1 year, accompanied by blurred vision for 7 months and numbness in his left face for 1 week was admitted to the Affiliated Hospital of Zunyi Medical University on May 7, 2022. One year ago, the patient had no obvious precipitating factor of paroxysmal stabbing pain in the whole skull with dizziness, which could be relieved by oneself after lasting for 1–2 min each time, with about 20 episodes per day. The cranial magnetic resonance imaging revealed changes in bilateral frontal lobe ischemic foci, bilateral frontal, ethmoid, sphenoid and maxillary sinusitis, and retinal macular degeneration. After hormone shock treatment, the condition improved. He suffered from headaches again with blurred vision in the right eye 7 months ago and was initially diagnosed with multiple sclerosis. He then was discharged after improvement due to hormone shock therapy. Oral hormone therapy was continued outside the hospital, but he stopped it due to drug side effects (details remained unclear). After cutting off, he developed a headache and visited our hospital once more, the relevant tests were performed and the patient was diagnosed with idiopathic hypertrophic pachymeningitis (IHP). The symptoms were slightly abated after hormone therapy. We hope that through this case report, we can deepen the clinicians' understanding of IHP, and improve the diagnosis rate of the disease through relevant examinations in future clinical work, so that patients can receive timely treatment and the mental pressure and economic burden caused by the disease on patients are reduced.

## Graphical abstract

We reported a case of idiopathic hypertrophic dura meningitis diagnosed in our hospital. The patient repeatedly suffered from headaches, followed by blurred vision in the right eye. During this period, multiple sclerosis was considered for diagnosis, and it improved after hormone treatment. However, the patient stopped taking the drug because of side effects; Later, he went to the hospital for treatment due to headache and blurred vision in his right eye again. After completing relevant examinations, the doctor considered to diagnose as idiopathic hypertrophic meningitis caused by anti‐myelin oligodendrocyte glycoprotein antibody. We hope that through this case report, clinicians will attach importance to hypertrophic pachymeningitis and its diagnosis and treatment.

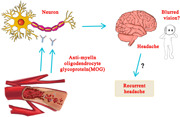

## 1 CASE INFORMATION

A 57‐year‐old man was admitted to the Affiliated Hospital of Zunyi Medical University on May 7, 2022, due to suffering from a headache for 1 year, accompanied by blurred vision for 7 months and numbness in his left face for 1 week. One year ago, the patient presented with a paroxysmal stabbing pain in the whole skull without any obvious incentive, accompanied by dizziness, which could be relieved by himself after lasting 1–2 min each time. The episodes occurred about 20 times a day. He had visited other local hospitals; magnetic resonance imaging (MRI) of his head showed changes in bilateral frontal lobe ischemic lesions, bilateral frontal, ethmoid, sphenoid and maxillary sinusitis, and macular degeneration of his retina, and his condition improved after receiving methylprednisolone 1000 mg pulse therapy.

The headache occurred again with blurred vision in the right eye 7 months ago, which was considered as multiple sclerosis. The patient was discharged after being improved by methylprednisolone 1000 mg impact treatment. Prednisone tablets were given outside the hospital at the initial dose of 60 mg/day, and then the drug was stopped due to drug side effects (the specific reason remained unclear). After cutting off the drug, the patient developed a headache again and presented to our hospital. After relevant examinations were performed, the patient was considered to have idiopathic hypertrophic pachymeningitis (IHP). His condition improved and then he was discharged after continuing with prednisone tablets.

## 2 PHYSICAL EXAMINATION ON ADMISSION

The indexes of patients are as follows: temperature at 36.4°C, pulse at 97 times/min, respiration at 20 times/min, and blood pressure at 115/64 mmHg. No obvious positive signs in the cardiopulmonary abdomen were observed. His consciousness was clear, his spirit was weak, his meningeal irritation sign was suspiciously positive, he had only light sensation in his right eye, and his visual acuity was decreased in his left eye; the pain on the left side of the face decreased obviously as compared with that on the right side. The left side was unable to lift up the soft palate, and the right side was normal. The extension of the tongue was left‐sided, and the strength of four limbs was grade 4. The tendon reflexes of both upper limbs were weakened, those of both lower limbs disappeared, and the physical examination of the remaining nerves was normal.

## 3 AUXILIARY EXAMINATIONS

Five items of thyroid function: thyrotropin of the third generation 0.396 μ IU/mL, triiodothyronine 0.96 nmol/L. Antinuclear antibody spectrum and antinuclear antibody (ANA) (1: 100) positive (nuclear granular and cytoplasmic granular), ANA (11: 320) positive (nuclear granular and cytoplasmic granular), ANA (I: 1000) weakly positive (nuclear granular + cytoplasmic granular). Anti‐ANCA‐GBM assay: anti‐GBM antibody 30.77R U/mL. Male gall‐associated antigens: ferritin 369.0 μg/L.

**Figure 1:**
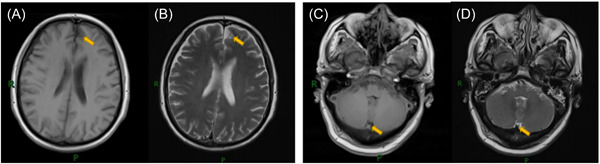
Brain magnetic resonance imaging scan + enhancement: bilateral frontal ischemic lesions (A, B). Bilateral parietal and tentorial meninges thickening and enhancement (C, D). The following possibilities are considered: intracranial hypotension syndrome, meningitis, idiopathic hypertrophic pachymeningitis, and so on. The pituitary gland is plump. [Color figure can be viewed at wileyonlinelibrary.com]

**Figure 2:**
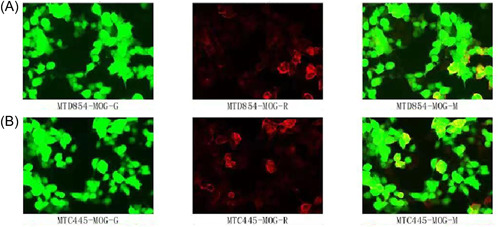
Cerebrospinal fluid (A) and serum (B) were positive for anti‐myelin oligodendrocyte protein antibodies by cytometric bead array (CBA) assay. [Color figure can be viewed at wileyonlinelibrary.com]

Nine joint inspections of respiratory pathogens result shown as follows: positive index of mycoplasma pneumoniae IgM. Electrocardiogram: no obvious abnormality is found; computed tomography (CT) scan of the chest: pneumonitis in both lungs, with pulmonary hemorrhage excluded. Brain MRI scan and enhancement: bilateral frontal ischemic lesions. Bilateral parietal and tentorial meninges thickening and enhancement. The following possibilities are considered: intracranial hypotension syndrome, meningitis, IHP, and so on. The pituitary gland is plump (Figure 1). To further improve the lumbar puncture technique, the color of cerebrospinal fluid was colorless and transparent, the pressure was 400 mm H_2_O, the routine cerebrospinal fluid setting was white blood cell count of 105 × 10^6^/L, and the biochemistry of cerebrospinal fluid setting was protein quantification of 1690 mg/L. External anti‐myelin oligodendrocyte glycoprotein antibody (serum): positive (titer 1:10). Anti‐myelin oligodendrocyte glycoprotein antibody (cerebrospinal fluid): positive (titer 1:10) (Figure 2, Table 1), and no obvious abnormality was observed for the rest.

**Table 1:** Differential diagnosis of demyelination of central nervous system (blood + cerebrospinal fluid).

Item	Method	Result	Reference value/range
Anti‐aquaporin 4 antibody (AQP4)	Cytometric bead array (CBA)	Negative (−)	Negative (−)
Anti‐myelin oligodendrocyte glycoprotein antibody (MOG)	CBA	**Positive (+) 1:10**	Negative (−)
Anti‐GFAP antibody	CBA	Negative (−)	Negative (−)
Anti‐myelin basic protein antibody (MBP)	CBA	Negative (−)	Negative (−)

*Note*: “Positive” means that we have detected MOG antibody in both the patient's serum and cerebrospinal fluid.

## 4 DIAGNOSIS

IHP is caused by anti‐myelin oligodendrocyte glycoprotein (MOG) antibody.

## 5 TREATMENT AND OUTCOMES

Oral prednisone was continued for the treatment, and mannitol and glycerofructose were dehydrated to reduce intracranial pressure to relieve the symptoms and pain. Other symptomatic treatments such as strengthening the brain, improving circulation and nourishing nerves relieved the headache of the patients, but they developed general weakness and vomiting, accompanied by cough after drinking water and dysphagia. Therefore, indwelling gastric tube was used to strengthen nutritional support. During hospitalization, patients who were accompanied with pulmonary infection, pulmonary hemorrhage and respiratory failure were given symptomatic treatments such as anti‐infection, phlegm reduction and oxygen therapy. In the end, the patient developed serious complications, so the therapeutic effect was poor.

## 6 DISCUSSION

Hypertrophic pachymeningitis (HP) is a rare neurological disease, and no large‐scale epidemiological study has been conducted yet. Its incidence rate is about 0.949/100,000.^1^, ^2^ Early symptoms of this disease are mild and nonspecific, which are easy to be missed and misdiagnosed. Failure to make timely diagnosis and treatment can lead to multi‐site involvement of the nervous system, which is prone to recurrence and has a poor prognosis.^3^

The etiology of HP is complex and diverse, with common causes including autoimmune diseases (e.g., rheumatoid arthritis), infections (e.g., tuberculosis, syphilis), systemic diseases (e.g., IgG4‐related diseases), tumors (e.g., lymphoma), and other confounding factors. Some HP with undetermined etiology is called IHP.^4^, ^5^, ^6^ Headache is the main clinical manifestation,^7^, ^8^ and cranial nerve defect is the second most common manifestation, with the optic nerve most commonly involved, followed by the cranial nerves in the III, IV, V, VI, and VIII pairs.^9^ Other clinical features include epileptic seizure, hearing loss, sensory impairment, dyskinesia, ataxia, and positive pathological reflex.^8^

Myelin oligodendrocyte glycoprotein antibody‐associated disease (MOGAD) is a rare autoimmune disease caused by MOG antibodies that can lead to demyelination of the central nervous system, mainly affecting the optic nerve, spinal cord, meninges, and brain stem.^10^, ^11^ Little is known with certainty regarding the pathogenesis of IHP caused by MOG antibody. Clinical presentation of IHP caused by MOG antibody is not distinguishable from other forms of hypertrophic pachymeningitis and reflects mechanical compression of vascular or nerve structures, leading to functional deficits.^6^

The specificity of early clinical symptoms of HP is poor, so perfecting multiple examinations plays a certain role in etiological diagnosis. Previous literature reports have confirmed the increase in inflammatory factors (high‐sensitivity C‐reactive protein and erythrocyte sedimentation rate). Nearly 70% of patients have intracranial hypertension and an increase in the number of leukocytes and protein level in cerebrospinal fluid.^7^, ^8^, ^11^ These patients have the above‐mentioned manifestations. Magnetic resonance contrast‐enhanced scanning is an important auxiliary examination mainly for evaluating the position, extent, and enhanced pattern of abnormal dura mater.^12^ Tissue biopsy, the gold standard for diagnosis of HP, shows connective tissue fibrosis and chronic inflammation, including infiltration of lymphocytes, plasma cells, and/or epithelioid histiocytes.^7^, ^8^, ^11^, ^12^ Unfortunately, no biopsy was performed in this case. However, the patient started with headache and gradually developed symptoms of cranial nerve damage in the development of the disease. Therefore, it was considered that this case was immune‐related idiopathic HP caused by MOG antibody in the final analysis based on the clinical manifestations and examination results.

For the treatment of HP, etiological treatment is the first choice for those with a clear source of infection (such as bacteria, fungi, tuberculosis, and viral etiology).^11^ Corticosteroids are the first choice for idiopathic HP when secondary factors are excluded. Steroid therapy can effectively alleviate the symptoms of idiopathic HP.^13^ A survey in Japan found that 87.2% of the 94 patients treated with corticosteroids had their symptoms effectively improved. For the patients who failed to respond to hormone therapy alone, after the combined use of immunosuppressive agents, the symptoms of about 92.6% of the patients improved.^14^ However, in some refractory cases, the patient may require surgical treatment. In our patient, clinical symptoms improved after corticosteroid shock therapy over a course of up to 1 year, suggesting that corticosteroids remained the first choice. However, disease recurrence is also one of the major problems in the treatment of hypertrophic meningitis. It has been reported that about 50% of patients with HP relapse after treatment, and the duration of relapse can range from 1 week to several years after initial treatment.^15^, ^16^ This was also confirmed in our patient, who had recurrent headache symptoms and presented with cranial nerve damage after hormone therapy.

In summary, the early diagnosis and treatment of HP are crucial for clinical prognosis. The recurrence rate of HP is high, and it is very important to start immunotherapy as soon as possible. MRI can be used to regularly evaluate the degree of nervous system damage and therapeutic effect in patients.

## AUTHOR CONTRIBUTIONS

Zhong Luo and Piao Cao conceptualized and designed the study, drafted the initial manuscript, and revised the manuscript. Jing‐Qing Xu and Jian Wang was responsible for data collection and revised the manuscript. Tao Liang and Rong Yan performed surgical treatment and revised the manuscript. Zu‐Cai Xu and Ya Chen developed a treatment plan, designed the data collection instruments, coordinated and supervised data collection, and critically reviewed the manuscript. All authors contributed to the interpretation of the findings and critical revision of the manuscript and approved the final manuscript. [Correction added on 12 February 2025, after first online publication: The authors surnames and given names have been updated as per their request.]

## CONFLICT OF INTEREST STATEMENT

The authors declare no conflict of interest.

## ETHICS STATEMENT

This study was approved by the Ethics Committee of ZunYi Medical University (Approval No. KLL‐2022‐773). The informed consent was signed by the patient.

## Data Availability

Data are available on request from the authors.

## References

[1] Yao A , Jia L , Wang B , Zhang J , Zhang J , Xu B . Idiopathic hypertrophic pachymeningitis mimicking meningioma with occlusion of superior sagittal sinus: case report and review of literature. World Neurosurg. 2019;127:534‐537. 10.1016/j.wneu.2019.04.006 30965168

[2] Yonekawa T , Murai H , Utsuki S , et al. A nationwide survey of hypertrophic pachymeningitis in Japan. J Neurol Neurosurg Psychiat. 2014;85(7):732‐739. 10.1136/jnnp-2013-306410 24273222

[3] Mazzurco M , Pavone P , Di Luca M , et al. Optic neuropathy, secondary to ethmoiditis, and onodi cell inflammation during childhood: a case report and review of the literature. Neuropediatrics. 2019;50(6):341‐345. 10.1055/s-0039-1693156 31330559

[4] El Aoud S , Frikha F , Ben Salah R , Snoussi M , Loukil H , Bahloul Z . Multiple cranial nerve palsy revealing hypertrophic pachymeningitis with positive myeloperoxidase‐antineutrophil cytoplasmic antibody. Reumatismo. 2013;65(5):248‐252. 10.4081/reumatismo.2013.248 24399188

[5] Charleston L , Cooper W . An update on idiopathic hypertrophic cranial pachymeningitis for the headache practitioner. Curr Pain Headache Rep. 2020;24(10):57. 10.1007/s11916-020-00893-5 32803475

[6] De Virgilio A , de Vincentiis M , Inghilleri M , et al. Idiopathic hypertrophic pachymeningitis: an autoimmune IgG4‐related disease. Immunol Res. 2017;65(1):386‐394. 10.1007/s12026-016-8863-1 27592235

[7] Bi Z , Shang K , Cao J , et al. Hypertrophic pachymeningitis in Chinese patients: presentation, radiological findings, and clinical course. BioMed Res Int. 2020;2020:1‐9. 10.1155/2020/2926419 PMC7448121 32879880

[8] Xiao X , Fu D , Feng L . Hypertrophic pachymeningitis in a southern Chinese population: a retrospective study. Front Neurol. 2020;11:565088. 10.3389/fneur.2020.565088 33281701 PMC7705170

[9] Hahn LD , Fulbright R , Baehring JM . Hypertrophic pachymeningitis. J Neurol Sci. 2016;367:278‐283. 10.1016/j.jns.2016.06.024 27423604

[10] Wu CS , Wang HP , Sung SF . Idiopathic hypertrophic pachymeningitis with anticardiolipin antibody: a case report. Medicine. 2021;100(2):e24387. 10.1097/md.0000000000024387 33466222 PMC7808507

[11] Papathanasiou A , Yeo JM , Humberstone M , Hosseini AA . MOG‐antibody‐associated hypertrophic pachymeningitis. Multiple Sclerosis Related Disord. 2020;42:102074. 10.1016/j.msard.2020.102074 32361264

[12] Oghalai JS , Ramirez AL , Hegarty JL , Jackler RK . Chronic pachymeningitis presenting as asymmetric sensorineural hearing loss. Otol Neurotol. 2004;25(4):616‐621. 10.1097/00129492-200407000-00033 15241244

[13] Zhu R , He Z , Ren Y . Idiopathic hypertrophic craniocervical pachymeningitis. Eur Spine J. 2015;24(suppl 4):633‐635. 10.1007/s00586-015-3956-4 25893340

[14] Huang Y , Chen J , Gui L . A case of idiopathic hypertrophic pachymeningitis presenting with chronic headache and multiple cranial nerve palsies: a case report. Medicine. 2017;96(29):e7549. 10.1097/md.0000000000007549 28723776 PMC5521916

[15] Zhao M , Geng T , Qiao L , et al. Idiopathic hypertrophic pachymeningitis: clinical, laboratory and neuroradiologic features in China. J Clin Neurosci. 2014;21(7):1127‐1132. 10.1016/j.jocn.2013.09.025 24589555

[16] Nicho N , Nozawa T , Murase A , et al. Difficulties of diagnosing idiopathic hypertrophic pachymeningitis in children: case report and literature review. Modern Rheumatol Case Rep. 2023;7(1):233‐236. 10.1093/mrcr/rxac026 35348716

